# Cold low-latitude Ordovician paleotemperatures may be in hot water

**DOI:** 10.1073/pnas.2424291122

**Published:** 2025-03-06

**Authors:** Ethan L. Grossman, Bryce B. Barney, Zeyang Sun, Gregory A. Henkes, Yang Gao, Michael M. Joachimski

**Affiliations:** ^a^Department of Geology and Geophysics, Texas A&M University, College Station, TX 77843; ^b^Department of Geosciences, Stony Brook University, Stony Brook, NY 11794; ^c^GeoZentrum Nordbayern, Friedrich-Alexander Universität Erlangen-Nürnberg, Erlangen 91054, Germany

Seawater’s oxygen-isotope (^18^O/^16^O) evolution is fundamental to oxygen-isotope (δ^18^O) paleothermometry and thus to understanding Earth’s climate, habitability, and biological evolution. Thiagarajan et al. (1) seawater δ^18^O in the Early Phanerozoic based on model-adjusted clumped and oxygen isotope analyses of fossils and especially fine-grained carbonate sediments. We are concerned, however, with the choice of fine-grained carbonate sediments as clumped and oxygen isotope archives, the assumption of closed-system diagenesis in the studied geologic units, and the geologically untenable cold paleotemperatures obtained. In addition, we believe that the paper is mistitled in that the reconstruction of Phanerozoic climate appears only in supplement.

## Choice of Fine-Grained Carbonate Sediments

The reliance on fine-grained carbonates with subordinate use of brachiopod shells, the standard for Paleozoic paleothermometry, is a concern. During lithification, the primary isotopic signatures of fine-grained Paleozoic carbonates are commonly overprinted by cementation and recrystallization of metastable phases like aragonite. For example, the studied Katian shallow-water carbonates have been interpreted as initially aragonite-dominated due to abundant green algae (2). Paleokarstic features point to subaerial exposure and diagenetic stabilization in meteoric waters (2). Both observations question the preservation of the original marine δ^18^O and clumped isotope paleotemperatures. Meanwhile, the information provided by the authors is not sufficient to support the preservation of the studied brachiopod shells.

## Assumption of Closed-System Diagenesis

The authors assume that the lowest clumped-isotope temperature (23 °C) and lowest seawater δ^18^O value (−4.0‰), both based on micrites sampled in outcrop, represent marine conditions and interpret other data within this framework. They state that “we are making the assumption that δ^18^O_seawater_ has a lower value than δ^18^O_water_ of diagenetic fluids” and further state that if diagenesis occurred in meteoric water, then their “model would not be able to distinguish between that and a low formation δ^18^O_seawater_ value” [Supplement, p. 3 (1)]. Considering the inevitability that platform carbonates will be exposed to meteoric water during sea level low-stands, and the evidence for meteoric diagenesis discussed above, we contend that the δ^18^O_water_ value of −4.0‰ reflects at least partial incorporation of freshwater δ^18^O. This hypothesis is consistent with the meteoric water δ^18^O expected for low latitudes if the hydrosphere δ^18^O remained relatively unchanged through the Phanerozoic (3).

## Geologically Untenable Cold Paleotemperatures

The paleoclimate dilemma of this paper is summarized in the authors’ figure 4, where temperatures have been calculated using the “new” seawater δ^18^O and published conodont apatite δ^18^O data from Estonia (4). Reconstructed temperatures for ~455 to 445 Ma are between 6 and 10 °C, incompatible with the Katian-age tropical carbonates with abundant stromatoporoids, corals, and green algae (e.g., dasycladaceans) as well as patch reefs (e.g., ref. 2). Equally implausible are results showing maximum tropical temperatures of 10 °C in the Carboniferous, when corals and coal swamps abounded.

**Fig. 1.**
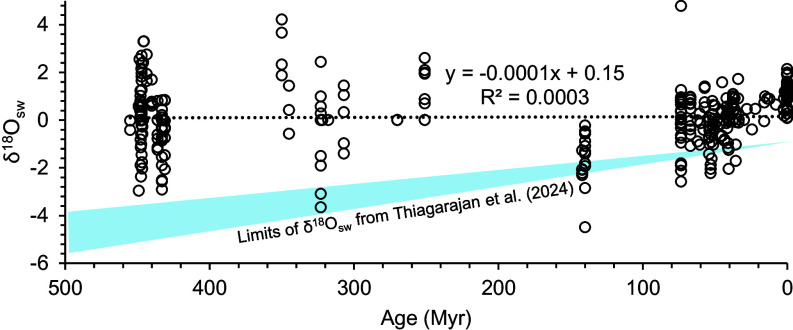
Calculated δ^18^O_seawater_ from clumped isotope paleotemperatures and δ^18^O values of carefully screen fossils (5–7). Also shown are similarly determined δ^18^O_seawater_ values derived from benthic foraminifera (8). The dotted line is a linear regression of the data, with a slope of ~0 ‰/Myr and an intercept that is consistent with constant Phanerozoic δ^18^O_seawater_. The blue field is the proposed limits of global seawater δ^18^O from Thiagarajan et al. (1) based on the model of ref. 9.

In contrast to Thiagarajan et al. (1), published records based on fossils and microfossils carefully screened for chemical alteration and clumped-isotope reordering show no change in the δ^18^O of seawater through time (Fig. 1). The consequence of this finding is that low-latitude Ordovician temperatures, rather than being cold, were warm or even hot (3).
